# Interactive effects of biochar and chemical fertilizer on water and nitrogen dynamics, soil properties and maize yield under different irrigation methods

**DOI:** 10.3389/fpls.2023.1230023

**Published:** 2023-09-06

**Authors:** Lei Wang, Shah Jahan Leghari, Jiajun Wu, Na Wang, Min Pang, Liang Jin

**Affiliations:** ^1^ Institute of Plant Nutrition, Resources and Environment, Beijing Academy of Agricultural and Forestry Sciences, Beijing, China; ^2^ College of Mechanical and Electronical Engineering, Northwest A&F University, Yangling, Shaanxi, China; ^3^ College of Resources and Environmental Sciences, Hebei Agriculture University, Baoding, China

**Keywords:** maize, biochar, water and nitrogen dynamics, border irrigation, drip irrigation

## Abstract

Long-term application of nitrogen (N) fertilizer adversely degrades soil and decreases crop yield. Biochar amendment with N fertilizer not only can increase yield but also can improve the soil. A 3-year field experiment was conducted to determine the effect of biochar doses with N fertilizer on maize yield and soil N and water dynamics under border irrigation (BI) and drip irrigation (DI) methods. Treatments were 260 kg N ha^−1^ without biochar addition and combined with low, medium, and high doses of biochar, namely, 15.5 t ha^−1^, 30.7 t ha^−1^, and 45.3 t ha^−1^ (NB_0_, NB_1_, NB_2_, and NB_3_), respectively. The biochar doses and irrigation methods significantly (*p* < 0.05) increased maize growth and yield characteristics, irrigation water use efficiency (IWUE), and fertilizer N use efficiency (FNUE) and enhanced the soil properties. In the BI and DI method, the NB_1_, NB_2_, and NB_3_ treatments increased yield by 4.96%–6.10%, 8.36%–9.85%, and 9.65%–11.41%, respectively, compared to NB_0_. In terms of IWUE and FNUE, the non-biochar treatment had lower IWUE and FNUE compared to biochar combined with N fertilizer treatments under both BI and DI methods. In the BI method, the IWUE in NB_2_ and NB_3_ ranged from 3.36 to 3.43 kg kg^−1^, and in DI, it was maximum, ranging from 5.70 to 5.94 kg kg^−1^. Similarly, these medium and high doses of biochar increased the FNUE of maize. The FNUEs in NB_2_ and NB_3_ under BI ranged from 38.72 to 38.95 kg kg^−1^ and from 38.89 to 39.58 kg kg^−1^, while FNUEs of these same treatments under DI ranged from 48.26 to 49.58 kg kg^−1^ and from 48.92 to 50.28 kg kg^−1^. The effect of biochar was more obvious in DI as compared to the BI method because soil water content (SWC) and soil N concentrations (SNCs) were higher at rhizosphere soil layers under DI. Biochar improved SWC and SNC at 0–20 cm and 20–40 cm soil layers and decreased below 60-cm soil layers. In contrast, despite biochar-controlled SWC and SNCs, still, values of these parameters were higher in deeper soil layers. In the BI method, the SNCs were higher at 60–80 cm and 80–100 cm compared to the top and middle soil layers. Depth-wise results of SNC demonstrated that the biochar’s ability to store SNC was further enhanced in the DI method. Moreover, biochar increased soil organic matter (OM) and soil aggregate stability and maintained pH. The NB_0_ treatment increased soil OM by 11.11%–14.60%, NB_2_ by 14.29%–19.42%, and NB_3_ by 21.98%–23.78% in both irrigation methods. This increased OM resulted in improved average soil aggregates stability by 2.45%–11.71% and 4.52%–14.66% in the BI and DI method, respectively. The results of our study revealed that combined application of N fertilizer with a medium dose of biochar under the DI method would be the best management practice, which will significantly increase crop yield, improve SWC, enrich SNC and OM, improve soil structure, and maintain pH.

## 1 Introduction

Maize is an important cereal crop that provides food to the world’s rising population (Haider et al., 2023). Maize contributes 35% of grain production in China (NBS, 2009) and consumes approximately 11.57b bushels (Taylor and Koo, 2013). Several factors can impact maize production, including soil fertility, water availability, and nutrient management practices (Kugedera et al., 2022). To increase grain yield, farmers apply excessive N fertilizer (Huang et al., 2021). However, the rate of N recovery from soil–plant systems is very low, hardly exceeding 50% of the applied fertilizer (Yang et al., 2020) due management practices (Geng et al., 2019). Soil condition has the greatest impact on N loss in agricultural systems (Bowles et al., 2018).

Nitrogen is a key component of chlorophyll, proteins, and nucleic acids, which are involved in various physiological processes, such as photosynthesis, respiration, and enzyme activity (Hassan et al., 2022). An adequate application of N fertilizer boosts vegetative growth, improves photosynthetic efficiency, and increases the grain yield of maize (Yue et al., 2021), while long-term N fertilizer use results in environmental pollution, particularly groundwater (Ju et al., 2006), surface water deterioration (Zhang et al., 2010), soil acidification (Schroder et al., 2011), and air pollution (Liu et al., 2011). In recent years, N fertilizer application increased (Du et al., 2018), with China’s agriculture system being the largest N fertilizer consumer (Tahir et al., 2021). The chemical fertilizer application in China has increased from 8.8 million tons in 1978 to 56.5 million tons in 2018, with chemical fertilizers used in China accounting for approximately 49% of total chemical fertilizer utilization worldwide (Chen, 2020). Usually, excessive N fertilizer is used to maximize crop yield in China, which surpasses crop N demand (Cui et al., 2010). Consequently, great amounts of N fertilizer losses via leaching and emissions (Khan et al., 2018). The nitrate form is highly water-soluble; therefore, it is easily leach down from the topsoil layer to bottom (Letey and Vaughan, 2013). Nitrate leaching accounts for approximately 18% to 20% (Ju and Zhang, 2017). Nitrate leaching adversely affects fertilizer N use efficiency (FNUE) of crop. FNUE commonly ranges between 30% and 35%. It is crucially important to improve FNUE by optimizing management practices, such as combining biochar with N fertilizer application.

Biochar, a multifunctional porous substance with a tiny particle size, a large surface area, a low bulk density, a high adsorption capacity, and an abundant carbon content, has received a great deal of attention because it provides numerous advantageous functions for crop production and soil science (Li et al., 2023). Biochar enhances soil fertility and nutrient availability, increases the efficiency of crop nutrient uptake (Gu et al., 2021), improves water retention, and reduces environmental risk (Yang et al., 2022). There are approximately 500 billion tons of biochar reserves in the world. It has been demonstrated to minimize ammonia emissions from animal dung composts while also improving compost quality (Li et al., 2020). Previous studies have shown that biochar decreased N leaching and emissions (Borchard et al., 2019) and improved N effectiveness for plant growth and development.

The combined application of N fertilizer and biochar has received a lot of interest because of its particular advantages in boosting soil N content (SNC) and improving FNUE (Xia et al., 2020). Mixed application of biochar with N fertilizer provided many benefits: (i) it helps in N nutrient release (Jeffery et al., 2017); (ii) its high cation exchange capacity, abundant pores, vast surface areas, and negatively charged surface improve electrostatic adsorption and retention of NH_4_^+^ (Mehmood et al., 2020); (iii) it has higher soil water-holding capacity (Farahani et al., 2020); (iv) it improves soil moisture at the topsoil layer, which helps in preventing N volatilization (Borchard et al., 2019); and (v) it improves soil microbe growth and N mineralization (Phares et al., 2022). The benefits of biochar for N adsorption and retention limit N losses due to volatilization and leaching, resulting in a progressive release of N for plant uptake and usage (Liu et al., 2021 *)* and high FNUE and crop yield (Phares et al., 2020). Also, biochar binds to micronutrients. Combined trace elements and minerals are more easily absorbed by plants. Biochar loosens compacted soil, thereby resulting in soil aeration and plant root growth. A meta-analysis study reported that biochar application with N fertilizer increases FNUE and crop yield by 10%–12.0% (Liu et al., 2022). However, the increase in FNUE and crop yield is also influenced by biochar doses, N fertilizer rates, and irrigation methods.

Border irrigation (BI) and drip irrigation (DI) are two commonly used irrigation methods in maize production systems. BI comprises flooding the field with water through border ridges, allowing the water to seep into the soil (Zhao et al., 2020). This method of irrigation is not controllable and does not provide uniform water distribution, resulting in maximum water loss and eroding N from the topsoil layer, but it is an easy and low-cost irrigation method compared to the DI method. On the other hand, the DI method is a precise and efficient method that delivers water directly and slowly to the root zone of plants through a network of drip lines (Fang and Su, 2019). The DI method is controllable, ensures optimal SWC, and decreases N loss from topsoil layers and nutrient uptake while ensuring focused water delivery, minimizing water loss from evaporation and runoff (Hallett et al., 2017).

The combined application of N fertilizer rates with different biochar dosages under BI and DI methods could have interactive effects on maize growth, nutrient uptake, and yield. The interaction between N and biochar may influence nutrient availability and water retention at different depths of the soil, thereby affecting plant growth and productivity. Yang et al. (2022) studied the effect of different doses of biochar on maize growth, yield and soil nutrient variation under the DI method and reported that increasing biochar amendment significantly improved the soil NPK availability, organic matter (OM) content, and maize growth and yield attributes. Previous studies have greatly focused on biochar and N fertilizer coupling effect on maize yield and soil indicators. Still, much work is required to understand the interaction between biochar doses and N fertilizer in the soil–plant system. We hypothesized that the combination of increased biochar doses with N fertilizer will improve soil and increase maize yield compared to a small dose of biochar. Therefore, the aim of this study was to investigate and compare the combined effects of biochar doses and N fertilizer rate on maize growth and yield, and SNC and SWC dynamics under BI and DI methods.

## 2 Materials and methods

### 2.1 Experiment site

**Figure 1:**
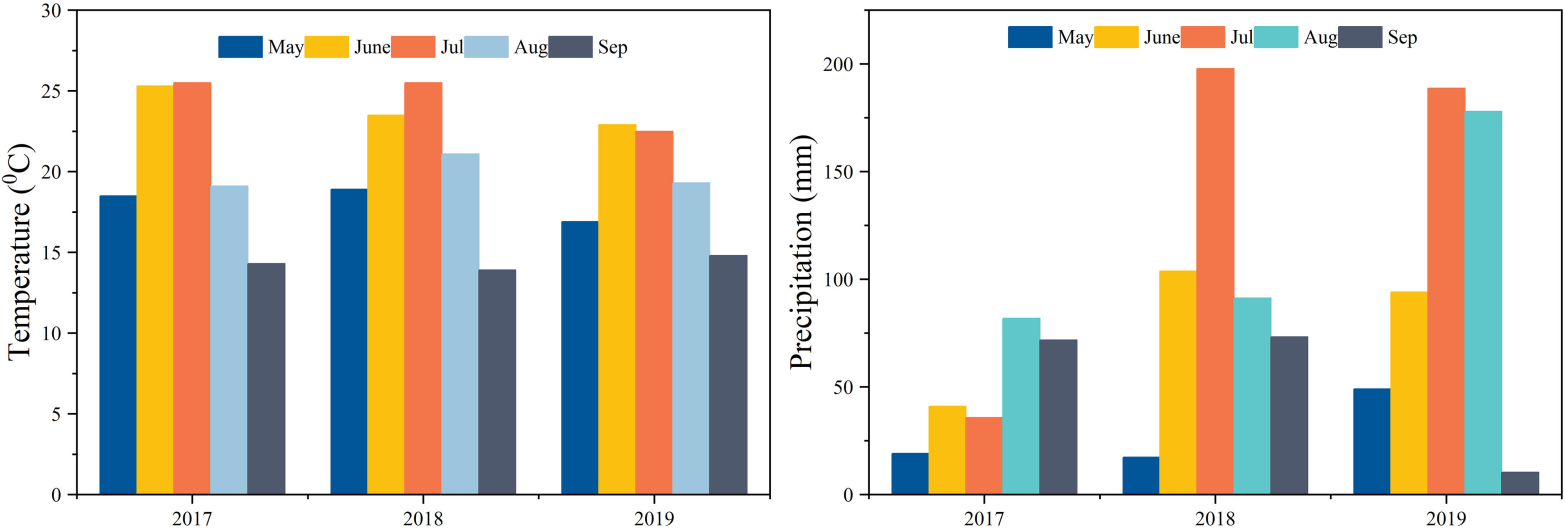
Temperature and precipitation during the maize growing season.

The experiment site is located in Qiqihar (47°21′15.65″ N latitude and 123°55′5.47″ E longitude), in the west-central part of Heilongjiang Province, northeastern China. The region has a temperate continental monsoon climate at a high altitude, spring is dry and windy, and summer is hot and rainy. Autumn is shorter, and winter is chilly and long, where the 24-h average summer temperature is 21.5°C, and the average annual rainfall is 410–540 mm; approximately 70% of rainfall occurs in the summer season. In the case of weather conditions during the present study, the rainfall was low in 2017 compared to 2018 and 2019. Rainfall mainly occurred in June and July as well as in August. The temperature and rainfall during the maize growing season are shown in **Figure 1**, and the basic soil properties are presented in **Table 1**.

**Table 1:** The soil properties at the experimental site.

Depth	BD	Particle fraction (%)			Soil texture (USDA)	pH	*θ_s_*	*θ_fc_*	*θ_wp_*	*K_s_*
(cm)	(g cm^–3^)	Sand	Silt	Clay			(cm^3^ cm^–3^)			(cm d^–1^)
0–20	1.33	17	25	58	Light clay loam	7.9	0.43	0.26	0.13	41.18
20–40	1.35	20	26	54	Light clay loam	7.8	0.40	0.23	0.14	33.19
40–60	1.32	22	27	52	Light clay loam	8.0	0.39	0.22	0.14	32.21
60–80	1.36	20	22	58	Light clay loam	7.6	0.38	0.21	0.15	49.11
80–100	1.36	21	22	57	Light clay loam	7.7	0.38	0.21	0.15	51.13

BD is soil bulk density, θ_s_ is soil saturated water content, θ_fc_ is field capacity, θ_w_p is soil water content at the permanent wilting point, and K_s_ is saturated hydraulic conductivity.

### 2.2 Experimental design

**Figure 2:**
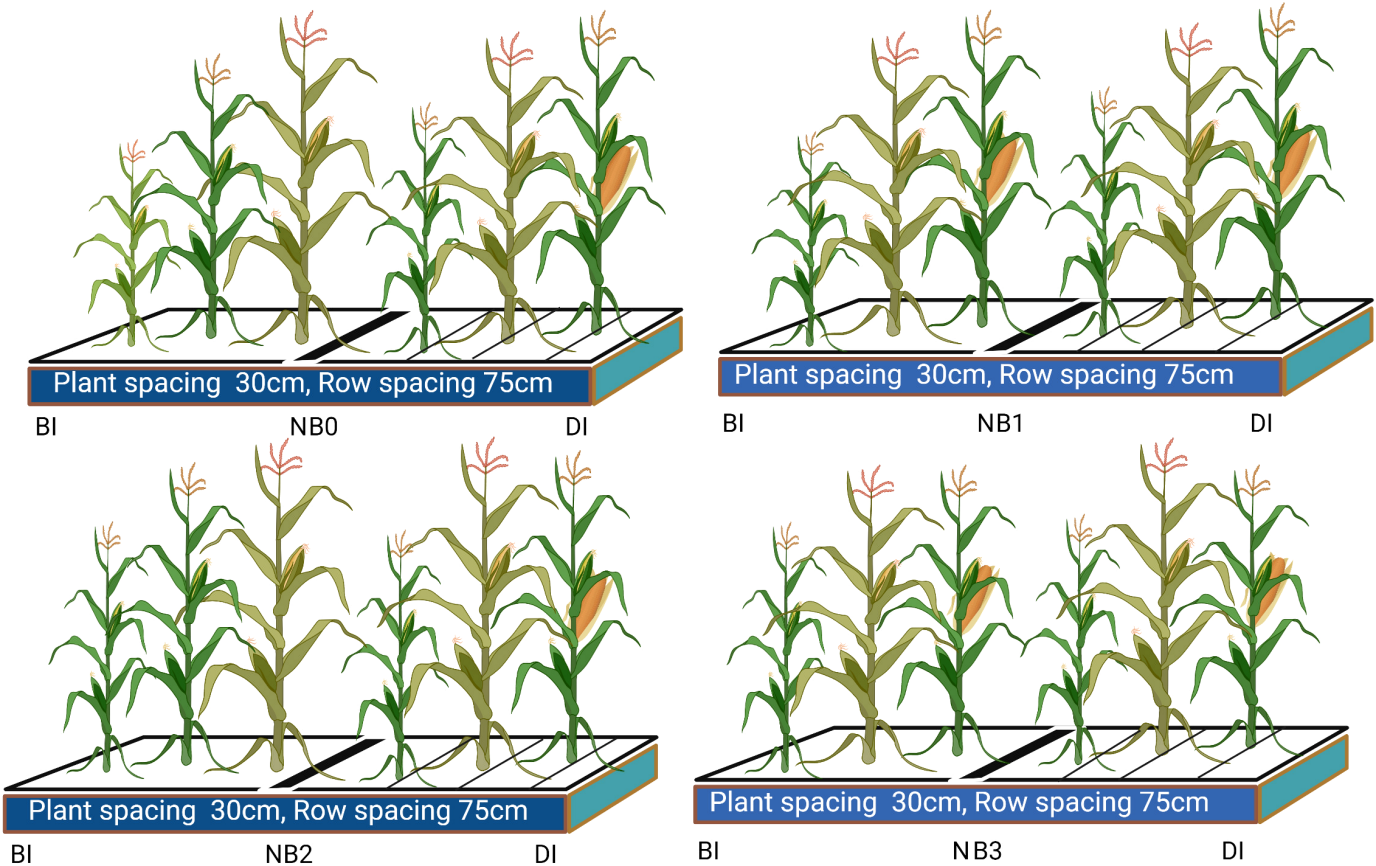
Cropping scheme. BI is the border irrigation method; DI is the drip irrigation method. NB_0_ is 260 kg N ha^−1^ without biochar amendment; NB_1_, NB_2_, and NB_3_ are combinations of N fertilizer with low, medium, and high doses of biochar, namely, 15.5 t ha^−1^, 30.7 t ha^−1^, and 45.3 t ha^−1^, respectively.

The field experiments were conducted from 2017 to 2019. Treatments were 260 kg N ha^−1^ alone and combined with low, medium, and high doses of biochar (B), namely, 15.5 t ha^−1^, 30.7 t ha^−1^, and 45.3 t ha^−1^ (NB_0,_ NB_1_, NB_2_, and NB_3_), respectively, under BI and DI methods. There were three replications. The maize variety was Nendan 19, and sowing dates were 7–9 May of each year. To achieve the recommended plant population of 67,000 ha^−1^, 28–30 kg·ha^−1^ seed was used. Seeds were sown in rows approximately 75 cm apart, and plant-to-plant spacing was 30 cm. The biochar used in this study was provided by Liaoning Biochar Engineering Technology Center and made from corn straw pyrolysis. It contained NPK 9.85 N, 1.63 P, 19 K, 55 Si, 2.9 Mg, 3.7 Ca, 163 O, and 595 OC g·kg^−1^. The basic pH of biochar was 7.5, and particle composition % was as follows: 14 (<0.1 mm), 61.3 (0.1–2 mm), and 25.1 (>2 mm). In the DI method, the irrigation amount was 220 mm and applied in small doses, with each application being 31.42 mm, while in the BI method, the irrigation amount was 300 mm and applied five times, with each application being 60 mm. In the DI method, the sub-main pipeline system supplied water to the laterals, while the lateral pipes on the plot carried water directly to the root zones of the crops. The inner diameter of the lateral pipes was 1.5 cm at a flow rate of 1.3 L h^−1^. The treatment plots were separated from adjoining plots by 1-m-wide isolation strips in order to account for the marginal effects of various irrigation techniques, and each plot (144 m^2^) was 18 m long and 8 m wide. In the experimental plot, the planting density was 7–8 plants/m^2^. The spacing between plants within a row was 14.4 cm, and the drip tapes were spaced apart by 110 cm. The plants were sowed in alternately wide and narrow rows, measuring 0.8 m and 0.3 m. A water reading meter was used to measure the irrigation water amount. To promote optimal seed germination and vigorous seedling establishment, the total dose of phosphorus (50 kg ha^−1^) and potassium fertilizers (250 kg ha^−1^) was incorporated into the soil during seedbed preparation. The source of phosphorus was monoammonium phosphate with an NPK content of 12:61:0, produced by Guizhou Kai Phosphorus Group Co., Ltd. (Guiyang, China). Potassium sulfate was used as the source of potassium, and its NPK ratio was 12:0:50, which is produced by Luobupo Potassium Salt Co., Ltd. (Xinjiang, China). To control the weed infestation in the experimental field, mechanical plough operation was performed between space of rows at the 3–5 leaf stage, and manual hand hoeing was performed at the 6–7 leaf stage. The cropping scheme is shown in **Figure 2**.

### 2.3 Field observations and measurement methods

The soil particle fraction was determined by using a hydrometer (Beretta et al., 2014). The soil water content was measured from soil depths of 0–120 cm at 20-cm intervals in each treatment plot of DI and BI using tubular FDR sensors (Zhang et al., 2020). Fresh soil samples were extracted with 1 mol L^−1^ KCl (1:10, soil/water) to estimate the soil mineral N concentration (SNC), which was then measured using a continuous flow analyzer (AA3, Bran and Luebbe, Germany). The leaf area index (LAI) was measured on different growth stages using a portable leaf area meter. When maize reached its full physiological maturity and grain moisture dropped below 13%, then it was harvested, and grain yields and dry matter (DM) were obtained. The irrigation water use efficiency (IWUE), fertilizer nitrogen use efficiency (FNUE), and yield changes were calculated as follows:(Si et al., 2020); (Qin et al., 2015); (Leghari et al., 2021)


(1) $$ET=P+I,\;WUE=\frac{Grain\;yield}{ET}\;IWUE=\frac{Grain\;yield\;(kg\;h^{-1})\;}{Amount\;of\;irrigation\;\left(mm\right)}$$

(2) $$FNUE=\frac{Grain\;yield\;(kg\;h^{-1})\;}{Amount\;of\;nitrogen\;fertilizer\;(kg\;h^{-1})}$$

(3) $$Yield\;change\;\left(\%\right)=\frac{Non-biochar_{NBO\;}-\;Biochar_{N+B1,N+B2\;andN+B3}}{Non-biochar_{NB0}}$$

where *I* is irrigation (mm), *P* is precipitation (mm), *ET* is evapotranspiration (mm), WUE is water use efficiency, IWUE is irrigation use efficiency, and FNUE is fertilizer N use efficiency. NB_0_ is 260 kg N ha**^−^** ^1^ without biochar amendment; NB_1_, NB_2_, and NB_3_ are combinations of N fertilizer with low, medium, and high doses of biochar, namely, 15.5 t ha**^−^** ^1^, 30.7 t ha**^−^** ^1^, and 45.3 t ha**^−^** ^1^, respectively.

### 2.4 Statistical analysis

The data were statistically analyzed using IBM SPSS Statistics 26. Analysis of variance (ANOVA) was used to determine differences between the control treatment, chemical fertilizer alone, and the interaction of biochar with chemical fertilizer on maize yield. The significant differences were detected at *p* < 0.05 using Duncan’s multiple ranges. Simple data processing was done in Excel and graphically visualized using Origin2021 software.

## 3 Results

### 3.1 Soil water content under the effect of biochar amendments with different irrigation methods

**Figure 3:**
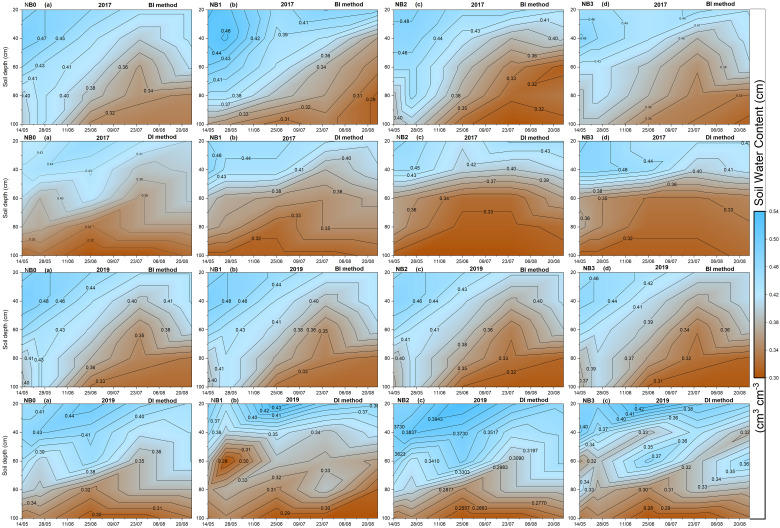
Temporal and spatial distribution of SWC in different depths under the effect of biochar. BI is the border irrigation method; DI is the drip irrigation method. NB_0_ is 260 kg N ha**^−^** ^1^ without biochar amendment; NB_1_, NB_2_, and NB_3_ are combinations of N fertilizer with low, medium, and high doses of biochar, namely, 15.5 t ha**^−^** ^1^, 30.7 t ha**^−^** ^1^, and 45.3 t ha**^−^** ^1^, respectively.

**Figure 3** shows the temporal and spatial distribution of SWC from the 0- to 120-cm soil profile under the effect of biochar with BI and DI methods. We measured soil water dynamics in the first and last seasons. In both seasons, it can be seen in **Figure 3** that SWC varied with biochar application levels, changing soil depths, and changing irrigation methods. A pattern of SWC distribution was totally different in the DI method as compared to the BI method. In the first season of the BI method, the SWC was increased below 60 cm depth. At the same time, it was decreased with the DI method. The DI method showed higher SWC in the topsoil layer and decreased in deeper soil layers. In non-biochar treatment NB_0_, the SWC was high at 60–80 cm and 80–100 cm soil depths under the BI method, and it was also higher in the DI method as well. All biochar-added treatments decreased ineffective SWC and maintained SWC at the optimum level. The NB_1_, NB_2_, and NB_3_ treatments under the BI method decreased SWC content at the 80[cm soil depth compared to NB_0_. The same treatments, NB_1_, NB_2_, and NB_3,_ under the DI method, decreased SWC at 60-cm to 100-cm soil depths. The biochar doses were high in NB_2_ and NB_3_ treatments; therefore, these treatments mainly enhanced SWC at 20-cm to 40-cm soil depths. Despite the high dose of biochar in NB_2_ and NB_3_ treatments restricting water from infiltrating in the deeper soil layers, water still moved into deeper soils under the BI method. However, this treatment showed a clear effect in the DI method. Considering the last season of the experiment, the SWC showed a different pattern of water distribution in the soil profile as compared to the first season. It could be because biochar does not decompose well in the early stage, so biochar particles absorb and store water. In the last season, still, the effect of biochar on different soil depths was positive under the DI method as well as the BI method. Biochar application also decreased ineffective SWC in the soil profile during the last season of the experiment, particularly in the deep soil layer. Maize is a shallow-rooted crop; therefore, it is critical that SWC content must be higher in the topsoil layer. Overall, experimental results related to SWC indicate that biochar application would be effective in BI and DI methods; however, more benefits of biochar can be achieved under the DI method as compared to the BI method.

### 3.2 Soil nitrogen concentration under the effect of biochar amendments with different irrigation methods

**Figure 4:**
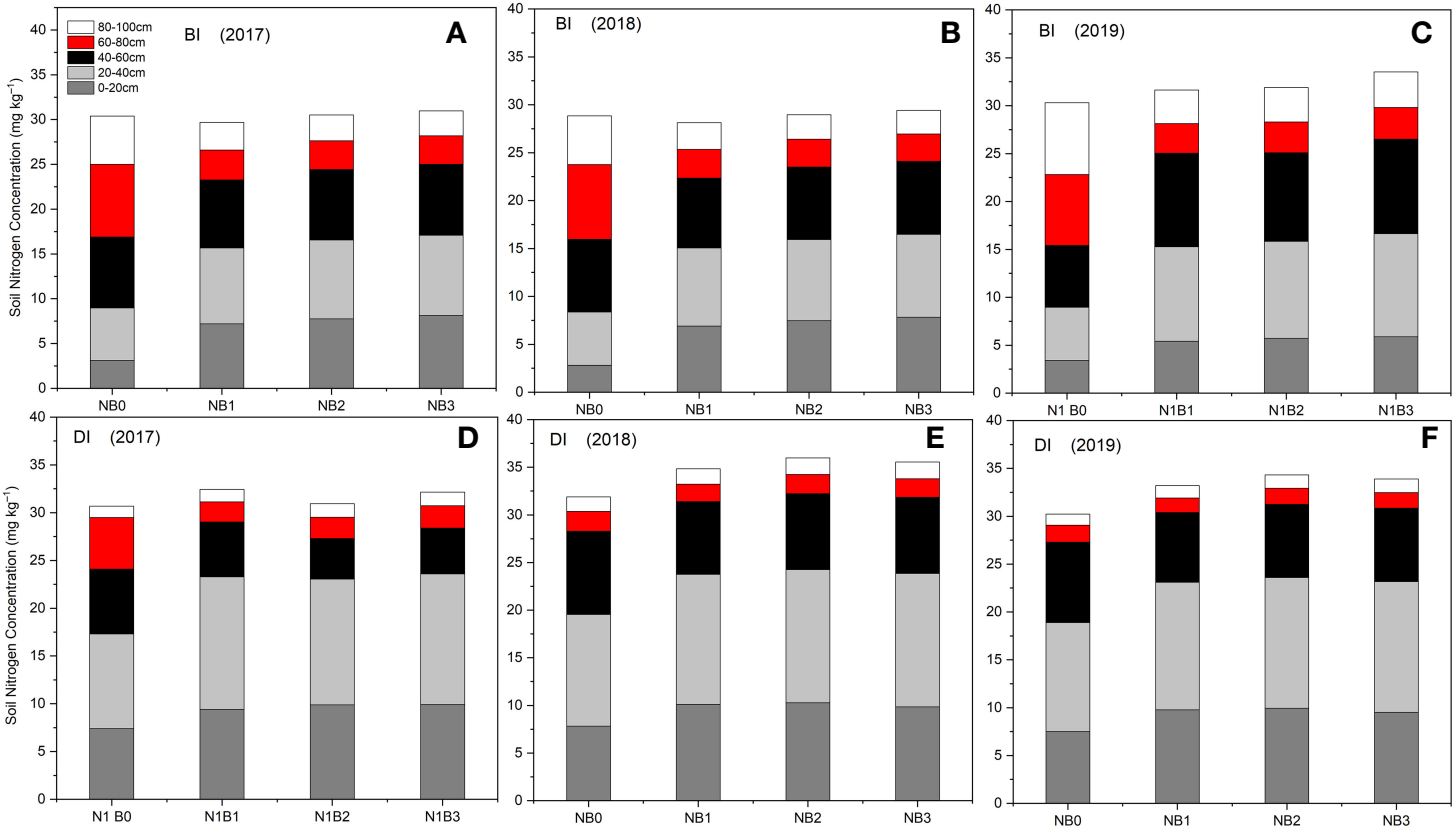
Distribution of SNC in different depths under the effect of biochar in 2017 **(A, D)**, 2018 **(B, E)** and 2019 **(C, F)**. BI is the border irrigation method; DI is drip irrigation method. NB_0_ is 260 kg N ha^−1^ without biochar amendment, NB_1_, NB_2_, and NB_3_ are combinations of N fertilizer with low, medium, and high doses of biochar, namely, 15.5 t ha^−1^, 30.7 t ha^−1^, and 45.3 t ha^−1^, respectively.

**Figure 4** shows the effect of biochar on SNC under BI and DI methods. As can be seen in **Figure 4**, the SNC varied with changing biochar doses, soil depths, and irrigation methods.

#### 3.2.1 BI method

In this irrigation method, the SNC at 0–20 cm, 20–40 cm, and 40–60 cm depths were significantly lower in non-biochar treatment NB_0_ and higher in 60–80 cm and 80–100 cm depths. This could be due to the maximum transportation of N along with water from the topsoil layers and accumulation in deeper soil layers. Despite the irrigation amount being high in the BI method, biochar holds N in upper soil layers in all treatments containing biochar sources. Biochar combined treatments, including NB_1_, NB_2_, and NB_3,_ resulted in maximum SNC at 0–20 cm, 20–40 cm and 40–60 cm depths and decreased SNC in 60–80 cm and 80–100 cm soil depths. This trend of N accumulation in soil was similar in the 2017, 2018, and 2019 seasons. However, SNCs were relatively higher in the last year of the experimental field. In the BI method, SNC level of NB_0_ ranged from 2.81 to 3.41 mg·kg^−1^ at 0–20 cm soil depth, from 5.86 to 5.55 mg·kg^−1^ at 20–40 cm depth, from 6.45 to 7.91 mg·kg^−1^ at 40–60 cm depth, from 7.41 to 8.12 mg·kg^−1^ at 60–80 cm depth, and from 5.06 to 7.50 mg·kg^−1^ at 80–100 cm soil depth. In NB_1_, NB_2_, and NB_3_ treatments, the SNC ranged from 5.41 to 8.13 mg·kg^−1^ at 0–20 cm soil depth, from 9.86 to 10.76 mg·kg^−1^ at 20–40 cm depth, from 7.27 to 9.86 mg·kg^−1^ at 40–60 cm depth, from 2.88 to 3.32 mg·kg^−1^ at 60–80 cm depth, and from 2.46 to 3.72 mg·kg^−1^ at 80–100 cm soil depth.

#### 3.2.2 DI method

In this irrigation method, compared to the BI method, the SNCs at 0–20 cm and 20–40 cm were significantly higher in all treatments, including non-biochar treatment, mainly SNCs in NB_1_, NB_2_, and NB_3_ treatments. Unlike the BI method, the DI method decreased SNC at 60–80 cm and 80–100 cm deeper soil layer depths. This could be because N did not transport along with water application from the topsoil layers and accumulated in topsoil layers because the amount of water in the DI method was significantly lower than in the BI method. Overall, results indicated that biochar holds maximum N in upper soil layers under the DI method. In all treatments, the SNC at 60–80 cm and 80–100 cm were substitutionally lower than in the BI method. The biochar’s ability to store SNC was further enhanced in the DI method. In the DI method, SNC in NB_0_ ranged from 7.41 to 7.84 mg·kg^−1^ at 0–20 cm soil depth, from 9.90 to 11.72 mg·kg^−1^ at 20–40 cm depth, from 6.79 to 8.72 mg·kg^−1^ at 40–60 cm depth, from 1.77 to 5.41 mg·kg^−1^ at 60–80 cm depth, and from 1.17 to 1.50 mg·kg^−1^ at 80–100 cm soil depth. In NB_1_, NB_2_, and NB_3_ treatments, SNC ranged from 9.41 to 10.27 mg·kg^−1^ at 0–20 cm soil depth, from 13.16 to 14.01 mg·kg^−1^ at 20–40 cm depth, from 4.26 to 7.99 mg·kg^−1^ at 40–60 cm depth, from 1.50 to 2.33 mg·kg^−1^ at 60–80 cm depth, and from 1.30 to 1.76 mg·kg^−1^ at 80–100 cm soil depth. Thus, experimental results related to SNC indicate that biochar application would be effective in both BI and DI methods; however, soil N holding efficiency of biochar under the DI method can be high compared to the BI method.

### 3.3 Soil organic matter, pH, and aggregate composition changes under the effect of biochar amendments with different irrigation methods

**Figure 5:**
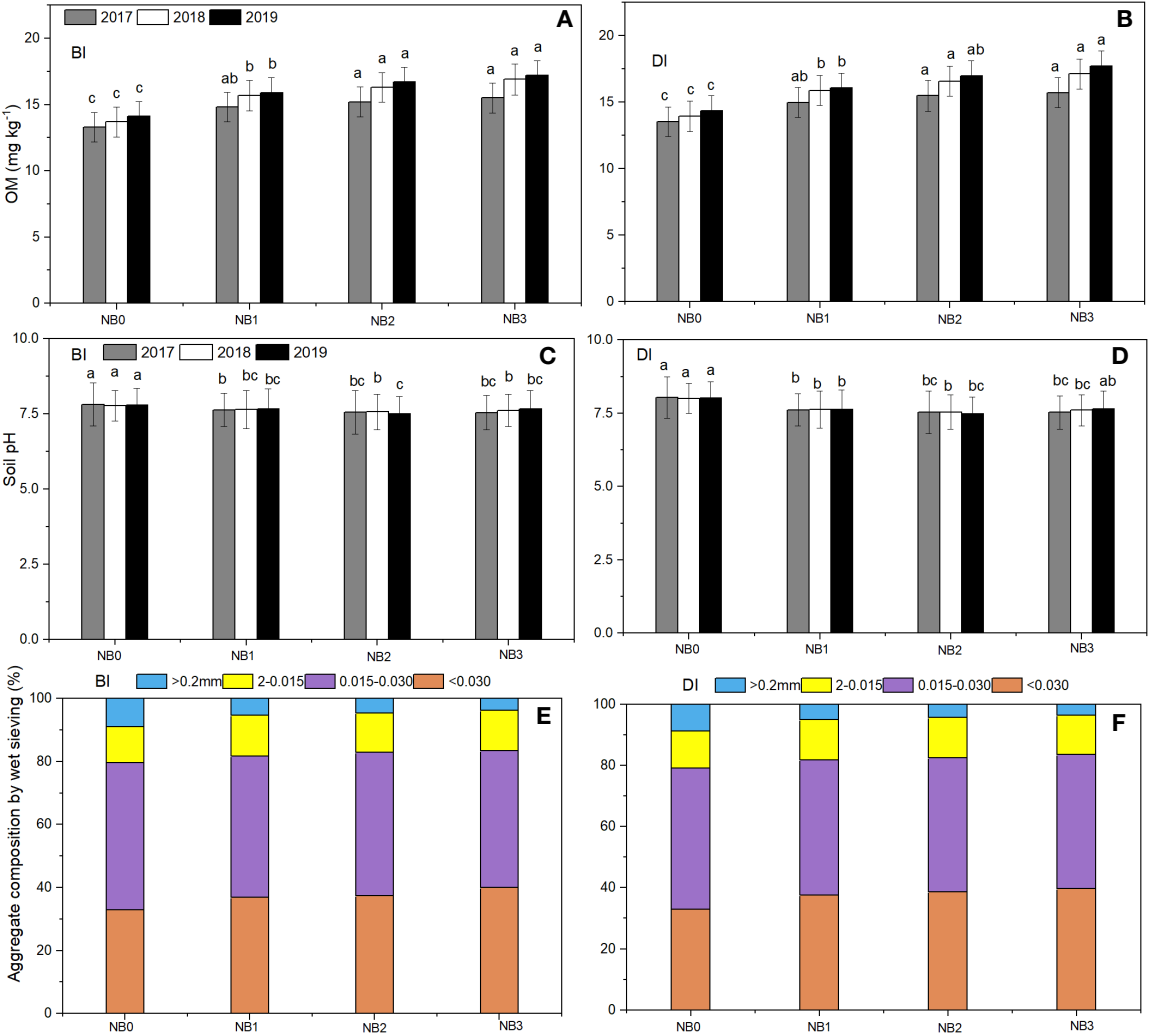
Soil OM, pH, and aggregate composition changes under the effect of biochar amendments. BI is the border irrigation method **(A, C, E)**; DI is the drip irrigation method **(B, D, F)**. NB_0_ is 260 kg N ha^−1^ without biochar amendment; NB_1_, NB_2_, and NB_3_ are combinations of N fertilizer with low, medium, and high doses of biochar, namely, 15.5 t ha^−1^, 30.7 t ha^−1^, and 45.3 t ha^−1^, respectively.

**Figure 5** shows the effect of biochar on soil OM and pH and Aggregate composition under BI and DI methods. The soil OM content varied with biochar application doses in both BI and DI methods. The soil OM content significantly increased with biochar amendment as compared to non-biochar treatment. The OM contents in NB_1_, NB_2_, and NB_3_ were significantly higher than in NB_0_. OM content gradually increased from the first season to the third season of the experiment. The OM contents were 13.3–14.3 g·kg^−1^, 14.8–16.1 g·kg^−1^, 15.2–17.0 g·kg^−1^, and 15.5–17.7 g·kg^−1^ in NB_0_, NB_1_, NB_2_, and NB_3_ treatments, respectively under BI and DI methods. Compared to non-biochar treatment under the BI method, NB_1_ increased OM by 11.28%–14.60%, NB_2_ by 14.29%–18.98%, and NB_3_ by 21.98%–23.36% from the first to the last season of the experiment. In the DI method, NB_1_ increased OM by 11.11%–14.39%, NB_2_ by 14.81%–19.42%, and NB_3_ by 16.30%–23.78%, compared to the NB_0_ treatment. This indicated that soil OM highly changes due to biochar application rates, but the irrigation method has some effect. The medium and high doses of biochar contributed largely to soil OM increments. The higher the biochar application rate, the higher the soil OM content, because biochar is a charred organic substance itself. Furthermore, the increase in OM content increased soil aggregate stability. Results showed that compared to non-biochar treatment, the NB_1_, N_1_B_2_, and N_1_B_3_ treatments improved the weight diameter of soil aggregates, and it is varied with irrigation methods as well. In the BI method, NB_1_, NB_2_, and NB_3_ increased the average soil aggregates stability by 2.45%, 4.01%, and 11.71%, and in the DI method, it increased by 4.52%, 8.19%, and 14.66%, respectively. In terms of soil pH, results revealed that biochar combination with N fertilizer could regulate soil pH under BI and DI methods. The NB_1_, NB_2_, and NB_3_ treatments significantly decreased soil pH compared to N fertilizer alone. In 3 years, NB_1_ decreased soil pH on average by 1.93%–4.99% and NB_2_ and NB_2_ by 2.44%–6.36%, compared to NB_0_ treatment under BI and DI methods. The pH, OM, and soil aggregate stability indicators indicated that biochar application would be crucial to improve soil properties.

### 3.4 Maize leaf area index under biochar amendments with different irrigation methods

**Figure 6:**
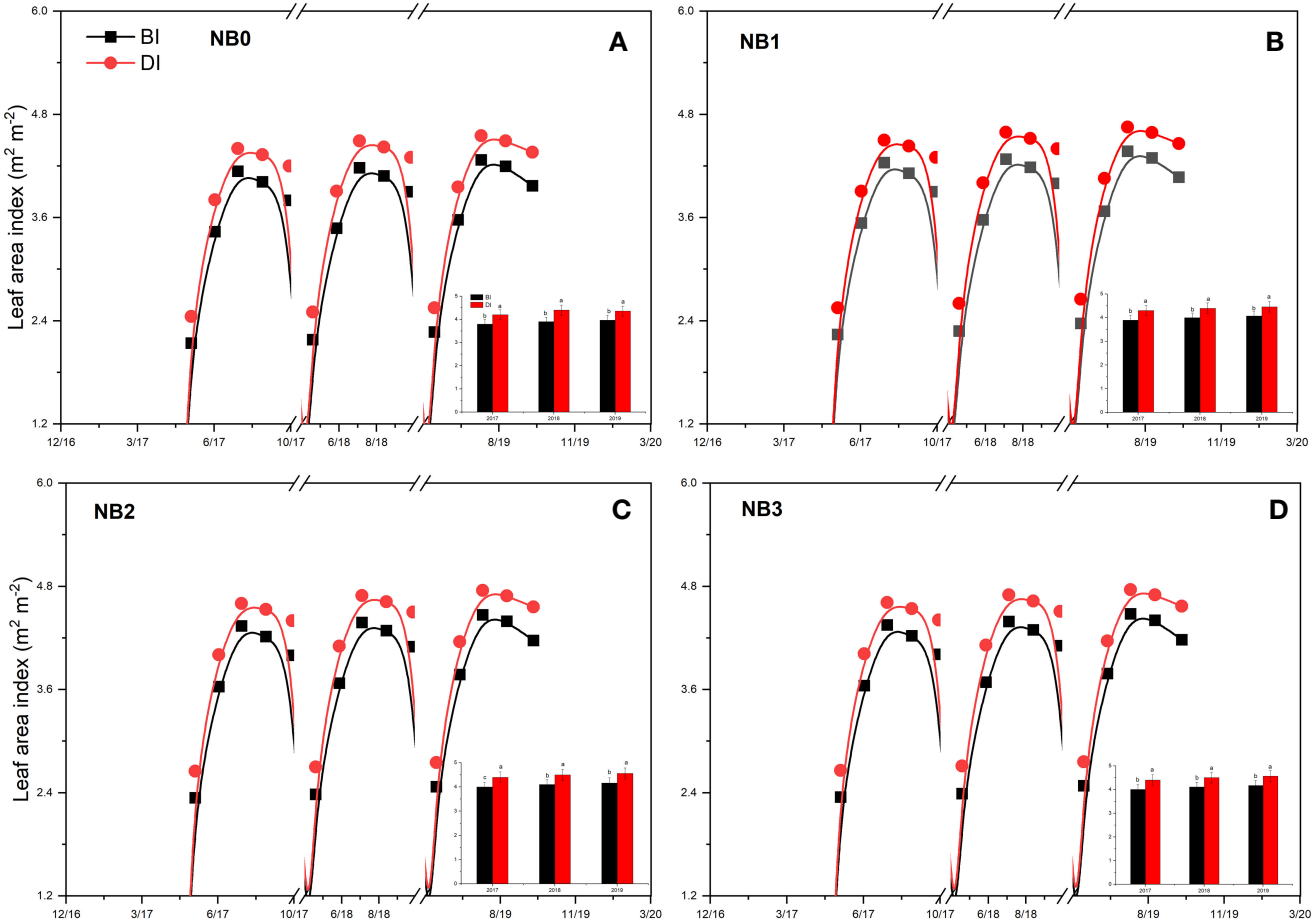
Maize LAI under the effect of biochar amendments. BI is the border irrigation method; DI is the drip irrigation method. NB_0_ is 260 kg N ha^−1^ without biochar amendment **(A)**; NB_1_, NB_2_, and NB_3_ are combinations of N fertilizer with low **(B)**, medium **(C)**, and high **(D)** doses of biochar, namely, 15.5 t ha^−1^, 30.7 t ha^−1^, and 45.3 t ha^−1^, respectively.

**Figure 6** shows the effect of biochar on the LAI of maize crops under BI and DI methods. It can be seen in **Figure 6** that irrigation methods have a significant (*p* < 0.05) effect on maize LAI development, and LAI development is also influenced by biochar doses, but the final LAI was not significantly different (*p* > 0.05). This could be due to the effect of rainfall or the maize plant recovered vegetative growth rate at the maturity stage, but it did not contribute to yield. The LAI was relatively larger during the second and third seasons as compared to the first season. It could be due to biochar increased nutrient availability in soil with time. The final LAI of maize in NB0 under the BI method was 3.80 m^2^ m^−2^, 3.90 m^2^ m^−2^, and 3.97 m^2^ m^−2^, and under the DI method, it was 4.20 m^2^ m^−2^, 4.40 m^2^ m^−2^, and 4.36 m^2^ m^−2^ in 2017, 2018, and 2019, respectively. The LAI of maize in NB_1_ under the DI method was 3.90 m^2^ m^−2^, 4.00 m^2^ m^−2^, and 4.07 m^2^ m^−2^, and under the DI method, it was 4.30 m^2^ m^−2^, 4.40 m^2^ m^−2^, and 4.46 m^2^ m^−2^ in 2017, 2018, and 2019, respectively. The maximum LAI of maize was in NB_2_ and NB_3_ under both BI and DI methods. The LAI of maize in NB_2_ and NB_3_ reached up to 4.56 m^2^ m^−2^. Compared to a maximum value of LAI in NB_0_ treatment under the BI method, the LAI of maize in NB_2_ and NB_3_ increased by 14%–15.11% under the DI method. Results demonstrated that the application of biochar could promote maize vegetative growth, and it could be clearly measured at different growth stages. In this regard, further research work is required.

### 3.5 Maize grain yields under biochar amendments with irrigation methods

**Figure 7:**
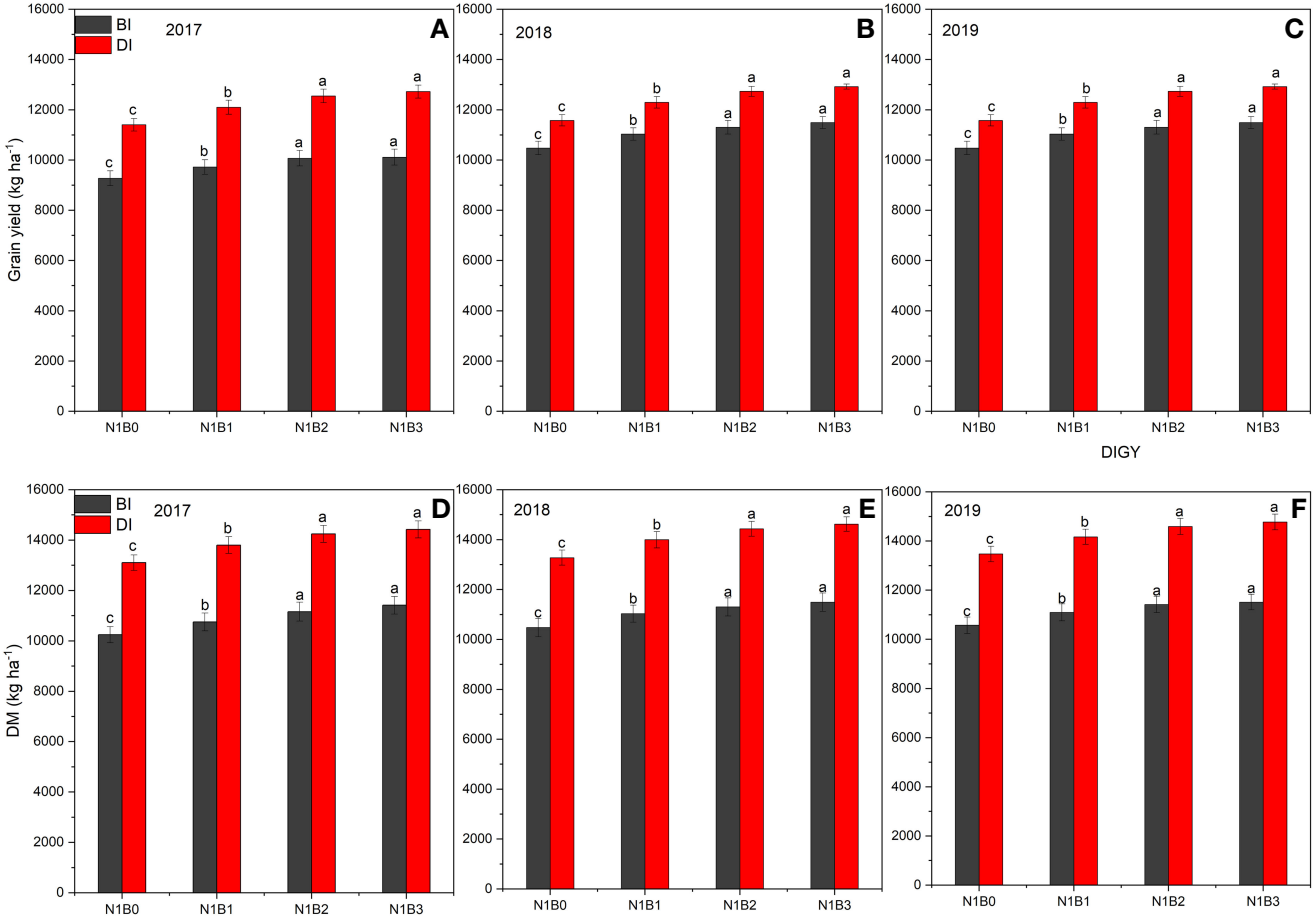
Maize grain yields under the effect of biochar amendmentsin 2017 **(A, D)**, 2018, **(B, E)**, 2019 **(C, F)**. BI is the border irrigation method; DI is the drip irrigation method. NB_0_ is 260 kg N ha**^−^** ^1^ without biochar amendment, NB_1_, NB_2_, and NB_3_ are combinations of N fertilizer with low, medium, and high doses of biochar, namely, 15.5 t ha**^−^** ^1^, 30.7 t ha**^−^** ^1^, and 45.3 t ha**^−^** ^1^, respectively. Different lowercase letters indicate significant difference at *p* < 0.05.

**Figure 7** and **Table 2** show the effect of biochar on maize grain yields and its characteristics under BI and DI methods. The irrigation methods and biochar doses have a significant effect on maize yield, IWUE, and FNUE at *p* < 0.05 and *p* < 0.01.

**Table 2:** Maize growth and yield attributes under the effect of biochar amendments.

Treatments	Methods	Grain yield (kg ha^−1^)				IWUE (kg kg^–1^)				FNUE (kg kg^–1)^			
		2017	2018	2019	Avg	2017	2018	2019	Avg	2017	2018	2019	Avg
NB_0_	BI	9,270f	9,337f	9,349e	9,319	3.09f	3.11f	3.12e	3.11	35.65f	35.91f	35.96e	35.84
NB_1_	BI	9,719e	9,800e	9,823de	9,781	3.24e	3.27e	3.27de	3.26	37.38e	37.69e	37.78de	37.62
NB_2_	BI	10,067d	10,100d	10,127d	10,098	3.36d	3.37d	3.38d	3.37	38.72d	38.56de	38.95d	38.84
NB_3_	BI	10,112d	10,025de	10,291d	10,218	3.37d	3.43de	3.43d	3.38	38.89d	38.85d	39.58d	39.30
NB_0_	DI	11,400c	11,576c	11,770c	11,582	5.18c	5.26d	5.35c	5.26	43.85c	44.52c	45.27c	44.55
NB_1_	DI	12,100b	12,293b	12,470b	12,288	5.50b	5.59b	5.67b	5.59	46.54b	47.28b	47.96b	47.26
NB_2_	DI	12,547a	12,732a	12,891ab	12,723	5.70a	5.79a	5.86ab	5.78	48.26a	48.97a	49.58ab	48.94
NB_3_	DI	12,720a	12,916a	13,072a	12,903	5.78a	5.87a	5.94a	5.86	48.92a	49.68a	50.28a	49.63
*F* value (M)		549.59**	541.11**	434.45**		39.57**	226.97**	36.78**		469.59**	441.11**	334.45**	
*F* value (B)		880.96**	677.65**	415.18**		12.42*	62.78*	13.41*		80.96*	77.65*	15.18*	
M×B		6.47**	5.60**	4.20**		8.88*	16.38*	0.01ns		3.47*	6.60*	0.42ns	
		Dry matter (kg ha^−1^)				Ear diameter (mm)				1,000-kernel weight (g)			
NB_0_	BI	10,242h	10,475f	10,571f	10,429	36.90cd	37.07de	37.89d	37.29	297.17d	300.46d	312.15c	297.17d
NB_1_	BI	10,750g	11,031e	11,093e	10,958	35.31d	36.11be	36.97d	36.13	301.53d	307.33d	309.27bc	301.53d
NB_2_	BI	11,157f	11,300d	11,417d	11,291	37.18c	37.90cd	38.05cd	37.71	309.19cd	317.12cd	321.33abc	309.19cd
NB_3_	BI	11,412e	11,489d	11,510d	11,470	38.15c	39.07c	39.82bc	39.01	313.39bcd	317.90bcd	321.54abc	313.39bcd
NB_0_	DI	13,100d	13,276c	13,470c	13,282	37.13cd	39.16c	39.97b	38.75	335.22b	337.23abc	339.21ab	335.22b
NB_1_	DI	13,800c	13,993b	14,170b	13,988	40.17b	43.14b	44.65a	42.65	340.67abc	342.45abc	345.37a	340.67abc
NB_2_	DI	14,247b	14,432a	14,591a	14,423	41.11ab	43.87ab	44.76a	43.25	343.18ab	346.14ab	346.13a	343.18ab
NB_3_	DI	14,420a	14,616a	14,772a	14,603	42.22a	45.19a	45.90a	44.44	344.11ab	347.14a	346.90a	344.11ab
*F* value (M)		6,150.26**	2,817.16**	2,815.23**		57.38**	189.82**	169.35**		26.82*	24.22*	17.67*	
*F* value (B)		205.75*	83.78*	73.42*		10.78*	19.96*	14.11*		0.23ns	0.94ns	0.42ns	
M×B		1.75ns	1.94ns	1.77ns		5.74*	8.11*	8.07*		0.33ns	0.09ns	0.15ns	
		LAI (m^2^ m^−2^)				Kernel number (per row)				Row number (per ear)			
NB_0_	BI	3.80b	3.90a	3.97a	3.89	24.29e	25.12f	25.80e	25.07	10.33c	10.57c	10.70c	10.53
NB_1_	BI	3.90ab	4.00a	4.07a	3.99	25.10cd	26.17e	26.90cd	26.06	10.55cb	10.77bc	10.80bc	10.71
NB_2_	BI	4.00a	4.10a	4.17a	4.09	26.33cd	27.23c	26.97cd	26.84	10.90ab	10.97ab	10.97b	10.95
NB_3_	BI	4.01a	4.11a	4.18a	4.1	27.19b	27.77b	28.09bc	27.68	11.01ab	11.17ab	11.19a	11.12
NB_0_	DI	4.20a	4.40ab	4.36a	4.32	25.51c	26.90c	26.95cd	26.45	10.75ab	11.01ab	10.90b	10.89
NB_1_	DI	4.30a	4.40ab	4.46a	4.39	26.79cd	27.30b	27.55bc	27.21	11.18b	11.89a	11.92a	11.66
NB_2_	DI	4.40a	4.50a	4.56a	4.49	28.13ab	28.79a	29.10ab	28.67	12.11a	12.17a	12.15a	12.14
NB_3_	DI	4.41a	4.51a	4.57a	4.5	28.85a	28.98a	29.90a	29.24	12.27a	12.55a	12.80a	12.54
*F* value (M)		3.23 ns	6.03*	7.46*		24.35*	705.74**	26.14*		9.34*	8.03*	7.92*	
*F* value (B)		2.20ns	1.02ns	0.16ns		92.59*	424.88*	15.92*		1.18ns	0.39ns	0.45ns	
M×B		0.00ns	0.99 ns	0.00ns		2.59*	8.09*	1.40ns		0.19ns	0.08ns	0.16ns	

BI is the border irrigation method; DI is the drip irrigation method. NB_0_ is 260 kg N ha^−1^ without biochar amendment; NB_1_, NB_2_, and NB_3_ are combinations of N fertilizer with low, medium, and high doses of biochar, namely,15.5 t ha^−1^, 30.7 t ha^−1^, and 45.3 t ha^−1^, respectively. IWUE is irrigation water use efficiency, FNUE is fertilizer nitrogen use efficiency, LAI is the leaf area index, M is irrigation method, and B is biochar dose. Different lowercase letters indicate significant differences at p < 0.05. *significant at p < 0.05, **significant at p < 0.01, and ns, non-significant.Discussion.

#### 3.5.1 BI method

In this irrigation method, the total maize productions of NB0, NB_1_, and NB_2_ treatments were 27,956 kg, 29,342 kg, and 30,294 kg, respectively, from 2017 to 2019. In t he NB_0_ non-biochar treatment, the maize yield was 9,270 kg ha^–1^ in 2017, 9,337 kg ha^–1^ in 2018, and 9,823 kg ha^–1^ in 2019. In NB_1_, the maize yield was 9,719 kg ha^–1^ in 2017, 9,800 kg ha^–1^ in 2018, and 12,470 kg ha^–1^ in 2019. In NB_2_, the maize yield was 10,067 kg ha^–1^ in 2017, 10,100 kg ha^–1^ in 2018, and 10,127 kg ha^–1^ in 2019. In NB_3_, the maize yield was 10,112 kg ha^–1^ in 2017, 10,251 kg ha^–1^ in 2018, and 10,291 kg ha^–1^ in 2019. Thus, on an annual average basis, NB_0_ had 9,319 kg ha^–1^, NB_1_ had 9,781 kg ha^–1^, NB_2_ had 10,098 kg ha^–1^, and NB_3_ had 10,218 kg ha^–1^. Compared to NB_0_, the NB_1_, NB_2_ and NB_3_ treatments increased maize yield by 4.96%, 8.36%, and 9.65%, respectively. Regarding IWUE, the non-biochar treatment NB_0_ had a lower IWUE compared to biochar doses combined with fertilizer. Maize IWUE in NB_0_ was 3.09 kg kg^−1^, 3.11 kg kg^−1^, and 3.12 kg kg^−1^ in 2017, 2018, and 2019, respectively, whereas maize IWUE in NB_1_ ranged from 3.24 to 3.27 kg kg^−1^. The NB_2_ and NB_2_ showed maximum IWUE, ranging from 3.36 to 3.43 kg kg^−1^. Considering the annual average IWUE of maize under different biochar treatments, the IWUE of NB_1_, NB_2_, and NB_3_ treatments increased by 5%–10% compared to non-biochar treatment. Similarly, biochar amendments also improved the FNUEs of maize crops; the maize had higher FNUE in NB_1_, NB_2_, and NB_3_ treatments. FNUE of the NB_0_ treatment ranged from 35.65 to 35.96 kg kg^−1^., whereas FNUE of the NB_1_ treatment ranged from 37.38 to 37.78 kg kg^−1^, FNUE of the NB_2_ treatment ranged from 38.72 to 38.95 kg kg^−1^, and FNUE of NB_3_ ranged from 38.89 to 39.58 kg kg^−1^. The order of yield, IWUE, and FNUE was NB_3_ ≥ NB_2_ > N_1_B_1_ > N_1_B_0_.

#### 3.5.2 DI method

In this irrigation method, the total maize production of NB_0_, NB_1_, NB_2_, and NB_3_ treatments were 36,863 kg, 36,863 kg, and 38,169 kg, respectively, from 2017 to 2019. In the NB_0_ non-biochar treatment, the maize yield was 11,400 kg ha^–1^ in 2017, 11,576 kg ha^–1^ in 2018, and 11,770 kg ha^–1^ in 2019. In NB_1_, the maize yield was 12,100 kg ha^–1^ in 2017, 12,293 kg ha^–1^ in 2018, and 12,470 kg ha^–1^ in 2019. In NB_2_, the maize yield was 12,547 kg ha^–1^ in 2017, 12,732 kg ha^–1^ in 2018, and 12,891 kg ha^–1^ in 2019. In NB_3_, the maize yield was 12,720 kg ha^–1^ in 2017, 12,916 kg ha^–1^ in 2018, and 13,072 kg ha^–1^ in 2019. Thus, on an annual average basis, NB_0_ had 11,582 kg ha^–1^, NB_1_ had 12,288 kg ha^–1^, NB_2_ had 12,723 kg ha^–1^, and NB_3_ had 12,903 kg ha^–1^. Compared to NB_0_, the NB_1_, NB_2_, and NB_3_ treatments increased yield by 6.10%, 9.85%, and 11.41%, respectively. In terms of IWUE, the non-biochar treatment NB_0_ had a lower IWUE compared to biochar combined with fertilizer treatments. Maize IWUE in NB_0_ was 5.18 kg kg^−1^, 5.26 kg kg^−1^, and 5.35 kg kg^−1^ in 2017, 2018, and 2019, respectively, whereas maize IWUE in NB_1_ ranged from 5.50 kg kg^−1^ to 5.67 kg kg^−1^. The NB_2_ and NB_2_ showed higher IWUE, ranging from 5.70 kg kg^−1^ to 5.94 kg kg^−1^. Considering the annual average IWUE of maize under different biochar treatments, the IWUE of NB_1_, NB_2_, and NB_3_ treatments increased by 6%–11% compared to non-biochar treatment. Similarly, biochar amendments also improved the FNUEs of maize crops; the maize had higher FNUE in NB_1_, NB_2_, and NB_3_ treatments. FNUE of NB_0_ treatment ranged from 43.85 to 45.27 kg kg^−1^, whereas FNUE of the N_1_B_1_ treatment ranged from 46.54 to 47.96kg kg^−1^, FNUE of the NB_2_ treatment ranged from 48.26 to 49.58 kg kg^−1^, and FNUE of NB_3_ ranged from 48.92 to 50.28 kg kg^−1^. The order of FNUE of maize under different treatments was the same as the BI method. However, in the DI method, the FNUE of maize was much higher than in the BI method. The order of yield, IWUE, and FNUE was NB_3_ ≥ NB_2_ > N_1_B_1_ > N_1_B_0_ in the DI method, whereas considering the irrigation method, the order of yield, IWUE, and FNUE was DI > FI.

## 4 Discussion

### 4.1 Effect of biochar on maize grain yields, IWUE, and FNUE under BI and DI methods

The fertilization and irrigation methods significantly affect maize yield, IWUE, and FNUE. Chemical N fertilizer alone was not efficient enough to increase yield. Biochar is a carbon-rich substance made from the pyrolysis of biomass. Its combined application with N fertilizer stimulates water retention capacity, water, and N uptake (Yang et al., 2022). Thus, it increases water and N productivity (Wu et al., 2021). The findings of this study revealed that biochar amounts improved maize yields, IWUE, and FNUE, and there were significant differences between the BI and DI methods at *p* < 0.05. The addition of low, medium, and high biochar doses 15.5 t ha^−1^, 30.7 t ha^−1^, and 45.3 t ha^−1^ with 260 kg N ha^−1^ increased maize yield by 4.96%, 8.36%, and 9.65% in the BI method, respectively. The benefit of biochar amendment was further enhanced in the DI method. The same combination of chemical fertilizer with biochar in the DI method increased maize yield by 6.10%, 9.85%, and 11.41%, respectively. These results are consistent with previous research that has shown the obvious impact of biochar with N fertilizer on crop yield (Jyrinki, 2022 *)*. Our results are also aligned with He et al. (2021), and Pathy et al. (2020) reported a significant increase in grain yields with the use of biochar. The positive effect of biochar on maize yield could be attributed to the following: (i) biochar enhances the soil texture and structure and microbial activity (Phares et al., 2022 *)*; (ii) it supplies minerals and soil organic carbon for plant growth (Liu et al., 2016 *)*; and (iii) it enhances soil nutrient storage, transformation, and absorption (Uzoma et al., 2011 *)*.

Regarding IWUE, results demonstrated that the N fertilizer application with biochar doses has a greater impact on maize IWUE than the non-biochar dose. Incorporating biochar into the soil can increase the IWUE of maize production systems. In the BI method, the IWUE varied from 3.09 to 3.12 kg kg^–1^ in NB_0_, while the N fertilizer with biochar doses (NB_1_, NB_2_, and NB_3_) showed higher IWUE, ranging from 3.24 to 3.43 kg kg^–1^. These results are consistent with Wu et al. (2022) and Ullah et al. (2020) studies; they reported an increased IWUE with the use of biochar and found that biochar maintained soil moisture by decreasing water loss via evapotranspiration, enhanced photosynthetic efficiency, and promoted chlorophyll synthesis and biomass accumulation. Similar to this, under the DI method, the IWUE varied from 5.18 to 5.35 kg kg^–1^ in NB_0_, while the N fertilizer with biochar doses exhibited higher IWUE, ranging from 5.50 to 5.94 kg kg^–1^. These findings are in line with previous studies that have highlighted the water-saving potential of biochar in DI systems. Qian et al. (2023) and Zheng et al. (2022) found an improved IWUE with the use of biochar in both BI and DI methods. The improved water-holding capacity of biochar-amended soils can also lead to reduced irrigation requirements, thereby improving IWUE. However, the specific effects may differ depending on factors such as biochar characteristics, soil properties, and environmental conditions.

Furthermore, the results exhibited that biochar treatments have higher FNUE compared to the non-biochar treatment under BI and DI methods. This indicates that the addition of biochar can increase N utilization in maize. In the BI method, the FNUE varied from 35.65 to 39.58 kg ha^–1^ in NB_0_, while the N fertilizer with biochar doses showed higher FNUE, ranging from 37.38 to 39.58 kg ha^–1^. These results related to FNUE are in line with previous research that has reported improved FNUE with biochar amendments (Guo et al., 2021 *;* Xu et al., 2023). Findings indicated that biochar application with N fertilizer increases plant N absorption and may be used as an N-releaser to efficiently supply sufficient substrates for plant growth and development (Ullah et al., 2021). The increase in plant N content is mainly linked to the use of biochar, which ensures more N is available to plants (Case et al., 2014) and enhances soil physicochemical qualities, such as water-holding capacity and bulk density, to provide ideal conditions for root development (Reibe et al., 2015). Under the DI method, the FNUE varied from 43.85 to 45.27 kg ha^–1^ in NB_0_, while the N fertilizer with biochar doses exhibited higher FNUE, ranging from 46.54 to 50.28 kg ha^–1^. These findings are consistent with Cao et al. (2019), and Tian et al. (2022) reported a significantly higher FNUE with the use of biochar, which improved soil nutrient cycling and reduced N losses. The following processes might explain boosting biochar’s effect on FNUE: (i) it binds N to create an agglomerated particle, preventing N_2_O emission and release of N (Shi et al., 2022), and (ii) it provides enough N by raising cation exchange capacity (Kumar et al., 2018). Overall results show that adding biochar into maize cropping systems, whether utilizing the BI or DI method, can result in higher maize yield, and increased IWUE and FNUE.

### 4.2 Effect of biochar on soil water and N dynamics under BI and DI methods

The SNC and SWC are crucially important in upper soil layers. The combined application of N fertilizer and biochar can boost upper soil fertility by decreasing nutrient leaching (Li et al., 2023). Biochar-amended soil water retention ability can enhance SWC by lowering water loss through evaporation and deep percolation under BI and DI methods (Zhao et al., 2020). Our findings suggest that biochar application can favorably alter soil water dynamics under various irrigation systems. The effect of biochar on SWC was observed to be significant, especially in the upper soil layers, and the pattern of SWC distribution differed between BI and DI methods. Results are consistent with earlier research studies that determined the impact of biochar on the water dynamics of soil. Liu et al. (2022) showed that biochar could improve soil water storage and infiltration, resulting in enhanced plant growth; Graber et al. (2010) reported that biochar could increase water retention capacity and decrease water loss due to evaporation, and Jeffery et al. (2011) found that biochar application can improve soil water-holding capacity, particularly in sandy soils. However, some studies have shown mixed results on the effect of biochar on soil water dynamics. For instance, Li et al. (2018) reported that the use of biochar did not significantly affect soil water retention capacity, although it did increase soil physical properties, and Major et al. (2010) found that the effect of biochar on SWC was highly dependent on biochar properties and soil type. The current study verified that adding biochar has a critical impact on soil water dynamics, especially in the DI method. The SWC varied with biochar doses and soil depths. Biochar doses under DI showed a higher SWC in the topsoil layer and decreased in deeper soil layers, and the effect of medium and high doses of biochar was obvious, which is consistent with the Dong et al. (2019) study. The results of the present study indicate that under BI, the SNC was lower in the non-biochar treatment in the upper depths and higher in the lower depths. This could be due to N fertilizer drained deep through the application of water, while the DI method decreased SNC at deeper soil layer depths. This suggests that N did not drain with water because of the low amount of irrigation application and biochar. These results agree with the finding of Gwenzi et al. (2016) who investigated the effects of different irrigation methods on SNC with the use of biochar and found that BI resulted in lower SNC at shallow depths but higher concentration at deeper depths, and Clough et al. (2013) studied the impact of biochar on N dynamics under different irrigation methods and found that the use of biochar decreased SNC in the upper soil layers for all irrigation methods. Overall, these results show that biochar stores the most N in the DI method, and this irrigation strategy also improves biochar’s capacity to store SNC. These results are related to the finding of Mehmood et al. (2020); the study found that biochar application increased SNC, reduced N leaching, and enhanced its use efficiency in maize crops. This can be attributed to the high cation exchange capacity and surface area of biochar, which promote N retention, and Lehmann et al. (2011) reported that biochar amendment enhanced accessibility in soil, resulting in improved N utilization of maize. Biochar’s porous nature makes it easier for water and nutrients to flow around, which improves water use effectiveness and decreases nutrient losses. The beneficial effects of biochar on water and N dynamics under various irrigation techniques are also demonstrated by these researchers. It is crucial to keep in mind that the specific impacts of biochar might change based on factors like the biochar feedstock, pyrolysis settings, soil characteristics, and crop varieties. In addition, various agricultural systems may require a varied biochar treatment rate and timing. Our findings support the concept that biochar can be a valuable soil amendment for improving soil moisture and nutrient status in topsoil layers.

### 4.3 Effect of biochar on soil OM, pH, and aggregate composition under BI and DI methods

Biochar has the ability to boost soil OM content by functioning as a long-term carbon sink in the soil as a stable form of organic carbon (Jeffery et al., 2011). Biochar can modify soil pH because of its alkaline nature, which can buffer acidic soils and potentially evaluate the pH level (Lehmann et al., 2011). However, depending on several factors, including the biochar composition, soil type, and irrigation methods, the specific impact of biochar on soil pH may change. The results indicate that the use of biochar had a major impact on the soil OM content when compared to the non-biochar treatment. The OM content of the soil increased with increasing biochar doses from low to high. Furthermore, the results also exhibited that the soil OM content gradually raised year after year. This shows that the benefits of biochar on soil OM may increase over time, resulting in even greater soil quality improvements, which agrees with Mehmood et al. (2020) findings. In all three seasons of the experiments, we saw a gradual rise in the soil OM content. The treatments with medium and high biochar application rates (30.7–45.3 t ha^−1^) highly increased OM content; the increase in soil OM content ranged from 11.11% to 23.78% in both BI and DI methods compared to the non-biochar treatment. These findings are consistent with previous works demonstrating that biochar amendments might increase soil OM accumulation. Yang et al. (2022) reported that the biochar additions increased soil OM content at the top 15 cm of soil by 14%–52% under different amounts of irrigation; Liu et al. (2021) reported that the biochar application rate of 20–60 t ha^−1^ increased soil OM by 2%–59%; and Dong et al. (2019) found an increase of soil OM by 18%–62%. The higher the biochar dose, the higher the OM content. The increased soil OM resulted in improved soil aggregate stability. Low, medium, and high doses of biochar increased soil aggregate stability to varying degrees. The NB_2_ and NB_3_ treatments increased the average soil aggregate stability by 4.01%–14.66% in both irrigation methods. Because soil structure is strangely related to OM. About a 3%–26% increase in soil aggregate stability and consistency is also reported by Blanco-Canqui (2017). In terms of soil pH, the findings showed that combining biochar with N fertilizer had a regulating impact on soil pH under both BI and DI methods. The NB_1_, NB_2_, and NB_3_ treatments resulted in a significant drop in soil pH compared to the NB_0_ treatment each year. The reduction in soil pH ranged from 1.93 to 6.36% in BI and DI methods. This finding suggests that biochar application with N fertilizer can help in pH adjustment, which is also confirmed by Phares et al. (2022), whereas N fertilizer application alone resulted in an increase in soil pH, which is consistent with the findings of Dong et al. (2022). Owing to its alkaline nature and significant potential to buffer soil pH, biochar seems to be a liming material in neutralizing the released protons in the event of N fertilizer application (Bolan et al., 2022). The findings of this study suggest that biochar application enhanced soil OM content substantially and medium and high biochar dosages have the largest impact on soil OM content. The data also demonstrate biochar’s regulating effect on soil pH when combined with N fertilizer.

## 5 Conclusion

In this study, the effect of different biochar doses on maize grain yields, N, and water dynamics was compared between conventional BI and DI methods. It was concluded that the application of medium (30.7 t ha^−1^) and high (45.3 t ha^−1^) doses of biochar could increase maize yield by 8.36%–9.65% and 9.85%–11.41% under BI and DI methods, respectively, compared to N fertilizer alone. Moreover, biochar increased FNUE and IWUE in both irrigation methods. Biochar application improved the SWC in topsoil layers. Results showed that in the BI method, the NB_0_ treatment decreased SNC at topsoil layers and increased at deep soil layers because N was transported along with a heavy application of water. When the irrigation method changed from BI to DI, the same treatment increased the SNC at the top layers and decreased it at deeper soil depths. The application of biochar doses further influenced the change in SNC. All combinations of N fertilizer with low, medium, and high biochar doses, namely, 15.5 t ha^−1^, 30.7 t ha^−1^, and 45.3 t ha^−1^, respectively, decreased N deposition in deeper soil layers. The SNC deposition in deeper soil layers under the DI method was much lower than in the BI method, indicating that biochar N holding efficiency in the topsoil layer was further enhanced in the DI method. Considering maize yield, FNUE, IWUE, and SNC dynamics, biochar application would be effective in the DI method, and a medium dose of biochar would be enough. Moreover, the effect of biochar was obvious on soil OM, soil aggerate stability, and regulation of pH. The soil OM increased with the application dose of biochar. Thus, it improved soil aggregate stability. The biochar application would be crucial to increase soil OM, improve soil structure, and maintain pH. In future studies, the long-term effect of biochar on soil properties should be studied with different N fertilizer application rates, and the optimal ratio of chemical fertilizer and biochar should be determined.

## Data availability statement

The original contributions presented in the study are included in the article/supplementary material. Further inquiries can be directed to the corresponding authors.

## Author contributions

LJ: funding acquisition, conceptualization, and supervision. JW: resources and project administration. LW and SL: investigation, writing—original draft, and methodology. LW, SL, NW, and MP: data collection. LW, SL, and JW: formal analysis. LJ, NW, and MP: writing—review and editing. All authors read and approved the final manuscript.
